# Dynamic Changes of the Blood Chemistry in Syrian Hamsters Post-Acute COVID-19

**DOI:** 10.1128/spectrum.02362-21

**Published:** 2022-02-23

**Authors:** Chi-Ju Hsu, Wen-Chin Lin, Yu-Ching Chou, Chuen-Mi Yang, Hsueh-Ling Wu, Yun-Hsiang Cheng, Ping-Cheng Liu, Jia-Yu Chang, Hsing-Yu Chen, Jun-Ren Sun

**Affiliations:** a Graduate Institute of Medical Sciences, National Defense Medical Center, Taipei, Taiwan; b Institute of Preventive Medicine, National Defense Medical Center, Taipei, Taiwan; c Graduate Institute of Pathology and Parasitology, National Defense Medical Center, Taipei, Taiwan; d School of Public Health, National Defense Medical Center, Taipei, Taiwan; e Department of Medical Techniques, Taipei City Hospital Ren-Ai Branch, Taipei, Taiwan; f Division of Infectious Diseases and Tropical Medicine, Department of Internal Medicine, Tri-Service General Hospital, National Defense Medical Center, Taipei, Taiwan; University of Manitoba

**Keywords:** SARS-CoV-2, blood chemistry, hamster

## Abstract

Severe acute respiratory syndrome coronavirus 2 (SARS-CoV-2) is a novel coronavirus that causes coronavirus disease 2019 (COVID-19). However, the long-term health consequences of COVID-19 are not fully understood. We aimed to determine the long-term lung pathology and blood chemistry changes in Syrian hamsters infected with SARS-CoV-2. Syrian hamsters (Mesocricetus auratus) were inoculated with 10^5^ PFU of SARS-CoV-2, and changes post-infection (pi) were observed for 20 days. On days 5 and 20 pi, the lungs were harvested and processed for pathology and viral load count. Multiple blood samples were collected every 3 to 5 days to observe dynamic changes in blood chemistry. Infected hamsters showed consistent weight loss until day 7 pi At day 5 pi, histopathology of the lungs showed moderate to severe inflammation and the virus could be detected. These results indicate that SARS-CoV-2 has an acute onset and recovery course in the hamster infection model. During the acute onset, blood triglyceride levels increased significantly at day 3 pi During the recovery course, uric acid and low-density lipoprotein levels increased significantly, but the total protein and albumin levels decreased. Together, our study suggests that SARS-CoV-2 infection in hamsters not only causes lung damage but also causes long-term changes in blood biochemistry during the recovery process.

**IMPORTANCE** COVID-19 is now considered a multiorgan disease with a wide range of manifestations. There are increasing reports of persistent and long-term effects after acute COVID-19, but the long-term health consequences of COVID-19 are not fully understood. This study reported for the first time the use of blood samples collected continuously in a SARS-CoV-2-infected hamster model, which provides more information about the dynamic changes in blood biochemistry during the acute and recovery phases of SARS-CoV-2 infection. Our study suggests that SARS-CoV-2 infection in hamsters not only causes lung damage but also causes long-term changes in blood biochemistry during the recovery process. The study may be used by several researchers and clinicians, especially those who are studying potential treatments for patients with post-acute COVID-19 syndrome.

## INTRODUCTION

Severe acute respiratory syndrome coronavirus 2 (SARS-CoV-2) causes coronavirus disease 2019 (COVID-19) and is highly infective via person-to-person transmission (1). SARS-CoV-2 infection has resulted in over 48 million cases, with more than 5.0 million deaths in 223 countries worldwide. COVID-19 has pushed the global health system to the brink of collapse. Antiviral interventions, including national blockades and social distancing, have severely disrupted global supply chains and the economy (2). Although COVID-19 is mainly a respiratory syndrome, patients experience several ongoing symptoms after recovery, including respiratory symptoms, tiredness, fatigue, and symptoms persisting for several months after the initial diagnosis (3). Post-COVID conditions are also known as post-COVID or long-COVID syndrome (4, 5). To date, the long-term effect of SARS-CoV-2 in humans remains poorly understood (6).

Several studies have demonstrated Syrian hamster (*Mesocricetus auratus*) as a small animal model that can be infected by SARS-CoV-2 (7–10). SARS-CoV-2-infected hamsters show respiratory symptoms, weight loss, and severe pathological lesions in the lungs, followed by pneumocyte hyperplasia (8). The infected hamsters naturally recovered within approximately 2 weeks after infection, similar to the mild course of human COVID-19 (11). This makes Syrian hamsters a particularly attractive animal model for evaluating the efficacy of SARS-CoV-2 vaccines and therapeutic candidates (10, 12).

Although the mortality rate of humans infected with SARS-CoV-2 is very low (about 2% to 5%), many hospitalized patients who have recovered from the infection develop post-COVID syndrome (4, 5). Many laboratories have reported pathological changes in Syrian hamsters during the course of infection, but their impact on blood chemistry is still unclear (8–11, 13). The main goal of this study was to evaluate the dynamic changes in blood chemistry to elucidate the long-term health effects of SARS-CoV-2 through multiple blood sampling strategies. Such information is also helpful for a more in-depth investigation of the mechanisms underlying post-COVID syndrome.

## RESULTS

### SARS-CoV-2 viral load and RNA copy numbers in infected hamsters.

**FIG 1:**
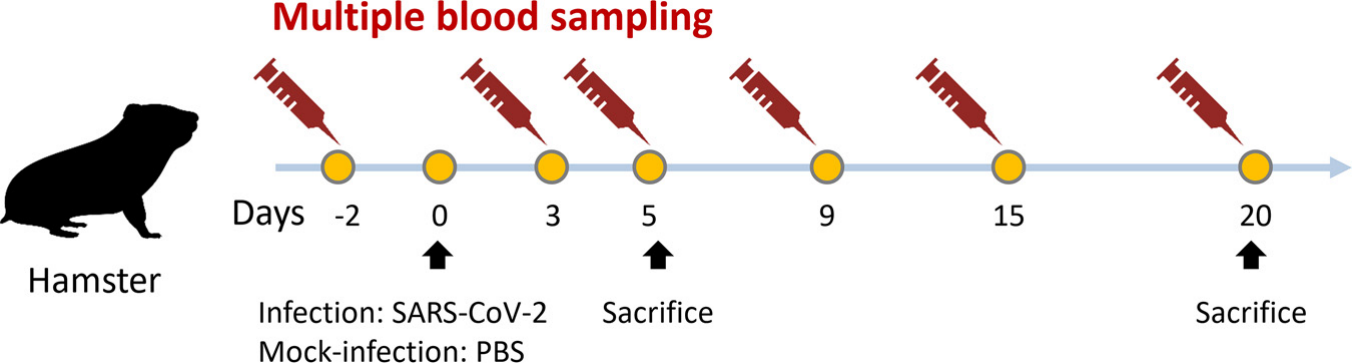
Schematic representation of timing of hamster infection, collection of multiple blood samples, and sacrifice for histological analysis and viral load analysis.

**FIG 2:**
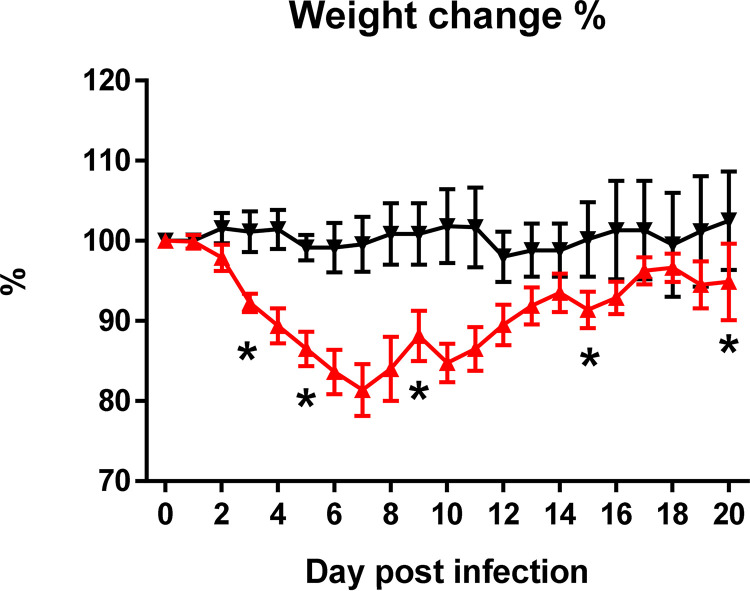
Body weight changes of the Syrian hamster model with 10^5^ PFU of SARS-CoV-2 infected (*n* = 8; red triangles) and mock-infected (*n* = 8; black inverted triangles) at days 0 to 20 (pi). *, *P* < 0.05.

Figure 1 shows the collection of blood samples at six time points. After the infection, the animals showed fatigue, lethargy, ruffled fur, and hunched back posture at day 2 postinfection (pi). The body weight of the infected hamsters decreased, becoming significantly different from that of the control animals at day 3 pi (mock versus SARS-CoV-2 infected; *P* < 0.05). At day 7 pi, weight loss was approximately 18.6%. Subsequently, the weight increased slowly every day until day 20 (Fig. 2). No infected or control animals died during the 20 days. The lung homogenates at day 5 pi had an average viral load of 8.7 ± 3.7 × 10^5^ PFU/mL. The average copies of nucleocapsid (NP) gRNA, ORF1ab region, envelope (*E*) gRNA, and *E* subgenomic RNA (sgRNA) were 6.0 × 10^6^ ± 5.7 × 10^6^, 8.6 × 10^5^ ± 7.9 × 10^5^, 3.4 × 10^6^ ± 2.9 × 10^6^, and 1.2 × 10^5^ ± 1.1 × 10^5^, respectively, per 10^5^ *GAPDH*. At day 20 pi, the virus was not recovered in any of the tree samples.

### Lung histopathology of infected hamsters.

**FIG 3:**
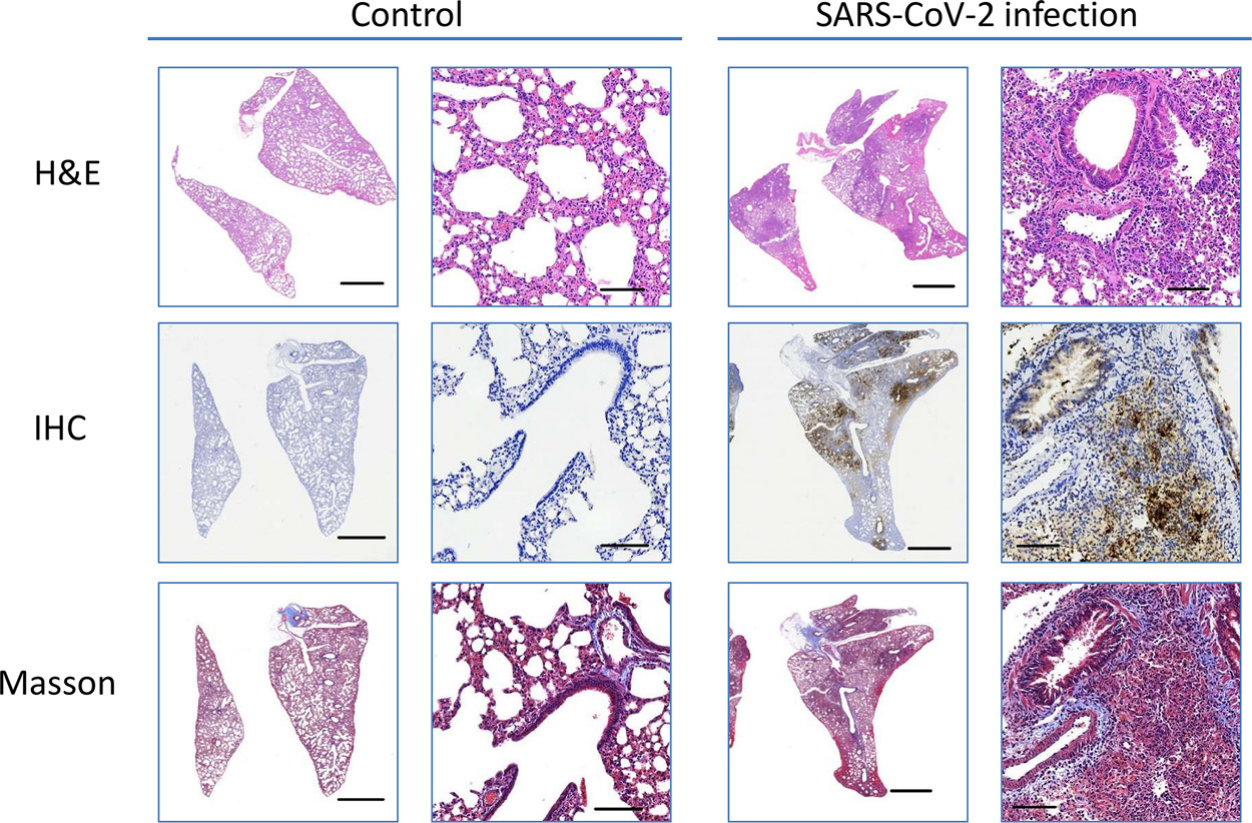
Macroscopic and microscopic view of the lungs at day 5 pi Scale bar for a closeup view is 2 mm. Scale bar for microphotograph is 100 µm.

**FIG 4:**
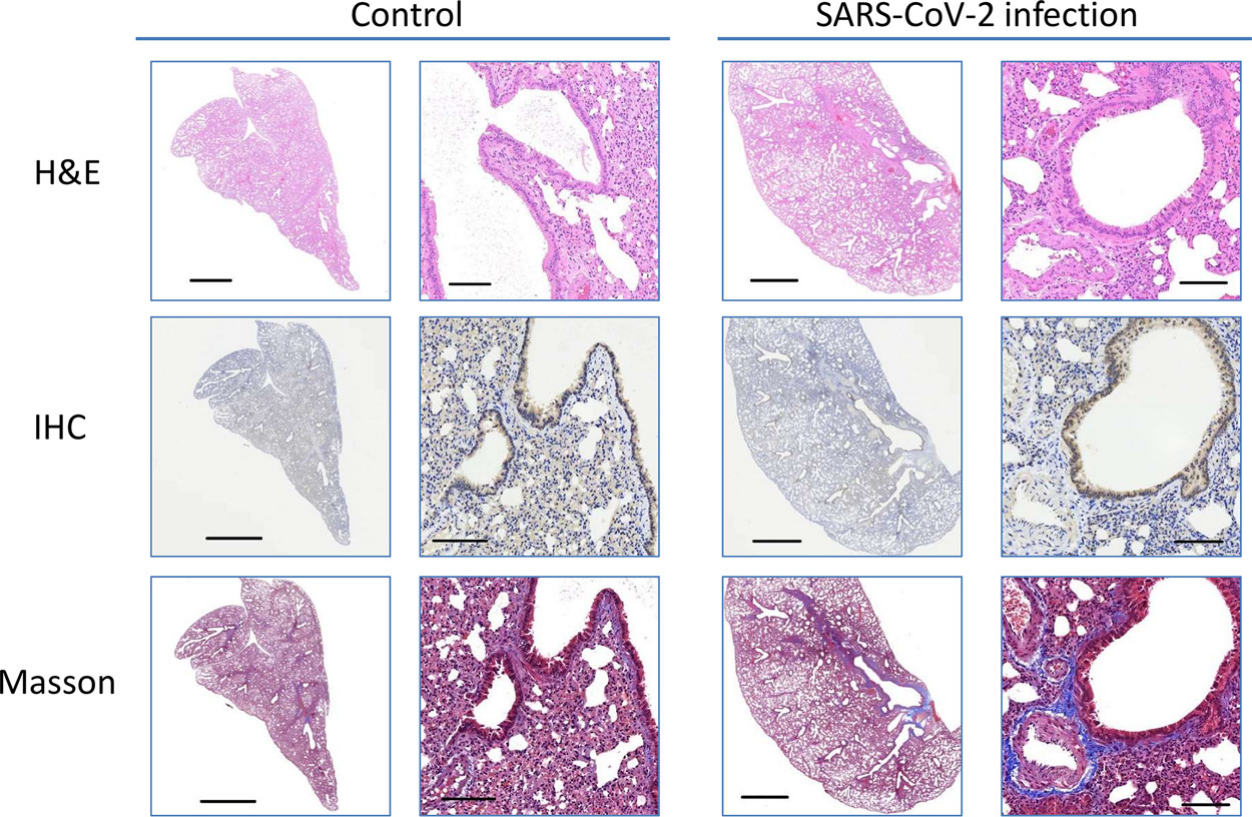
Macroscopic and microscopic view of the lung at day 20 pi Scale bar for a closeup view is 2 mm. Scale bar for microphotograph is 100 µm.

On days 5 and 20 pi, the lungs were examined for histopathology using hematoxylin and eosin (H&E), immunohistochemistry (IHC), and Masson’s staining (Fig. 3 and 4). Infected hamsters developed moderate to severe pulmonary inflammation. Pulmonary multifocal inflammation was characterized by histiocytes, neutrophils, and lymphocytes within the alveolar, interstitial, peribronchial, and perivascular areas at day 5 pi Pronounced interstitial and perivascular edema were detected, and pulmonary endothelialitis/vasculitis, which are considered important lesions in the hamster model, were observed consistently. Diffuse lesions in the lungs indicated a plateau period of infection. Contrarily, pulmonary inflammation and vascular lesions were observed in infected hamsters at day 20 pi, but the severity of the lesion was relatively low or absent. Recovery was noted as indicated by infected hamsters exhibiting mild type II alveolar epithelial cells. Meanwhile, IHC staining of N protein showed strong diffused expression in the infected lung at day 5 pi and diminished at day 20 pi, which concurred with the histopathological observations at day 20 pi However, moderate collagen was noted in Masson’s staining at days 5 and 20 pi

### Dynamic changes in blood biochemical markers in infected hamsters.

**FIG 5:**
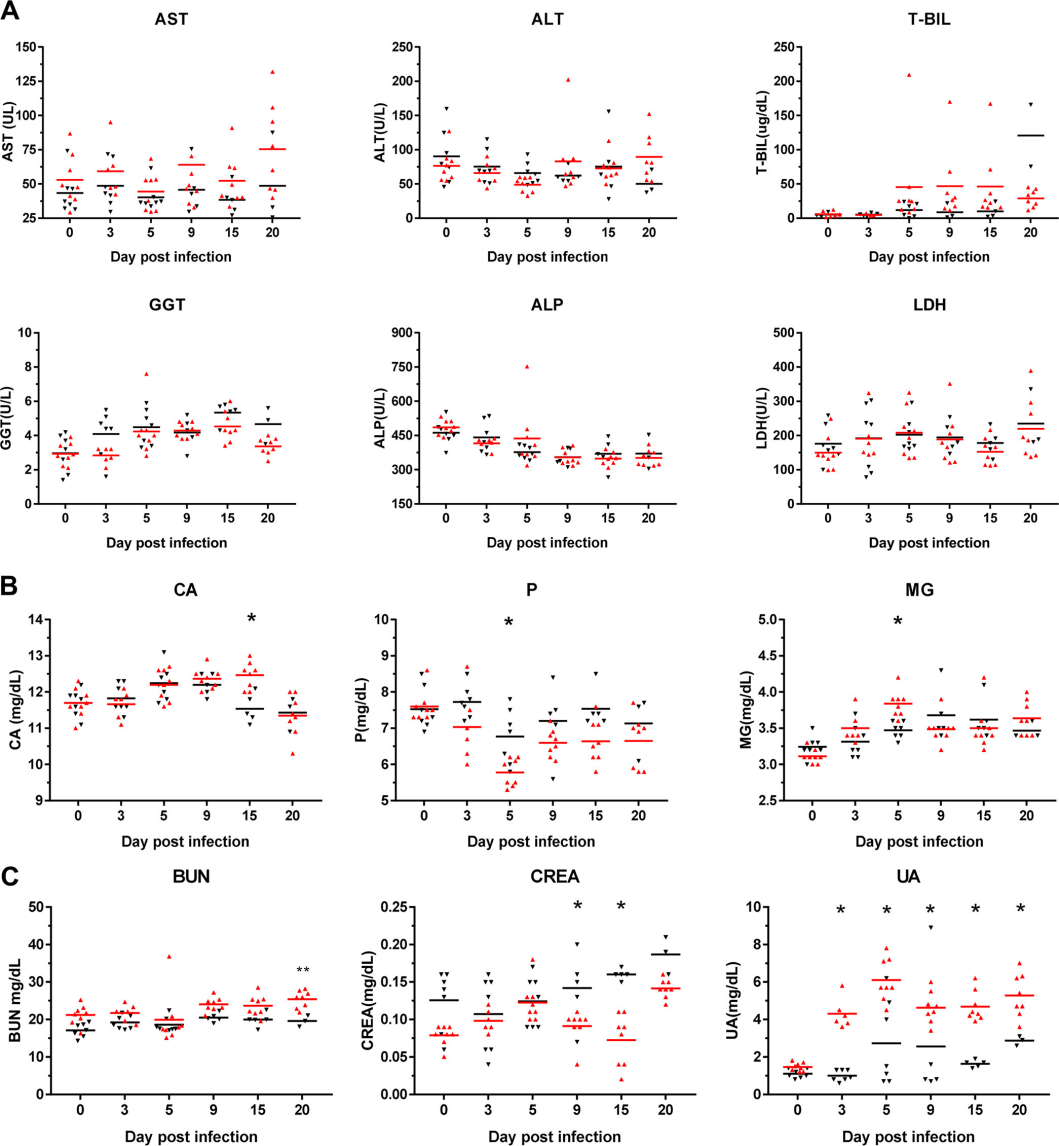

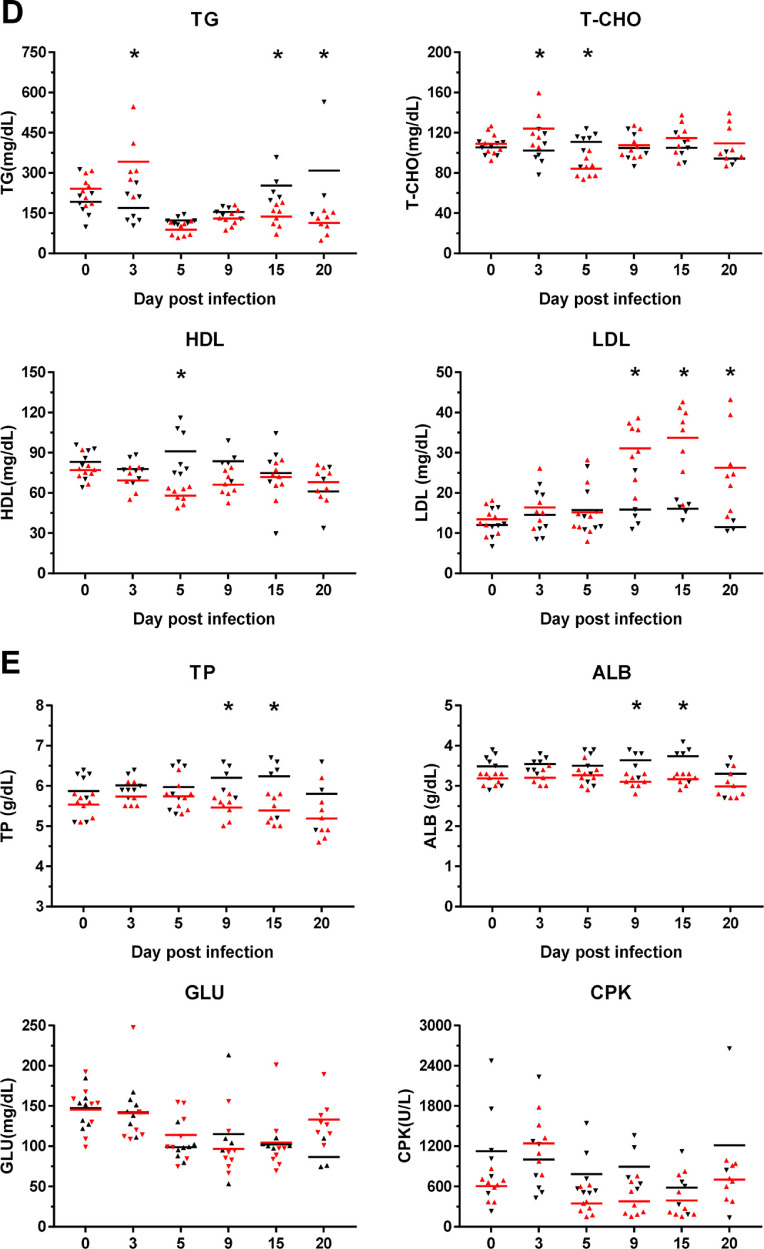
Alterations in the blood chemistry in SARS-CoV-2-infected hamsters. The means of the serum biomarker levels were noted, as indicated by a horizontal bar. (A) Liver biochemical markers, (B) blood mineral elements, (C) kidney biochemical markers, (D) blood lipids, and (E) energy-related markers. Syrian hamsters are infected with SARS-CoV-2 (red triangle) and mock infection (black inverted triangle). *, *P* < 0.05.

In this study, several serum biochemical markers were continuously monitored to analyze their relationship with infection. Compared with those of the control group, the infection group showed no significant continuous changes in hepatitis-related markers and blood mineral elements (Fig. 5A and B). In markers of renal disease and blood lipids, the levels of uric acid (UA), triglyceride (TG), and low-density lipoprotein (LDL) showed significant changes with time pi compared to those in the noninfected control (Fig. 5C and D). In energy-related markers, total protein (TP) and albumin (ALB) levels significantly decreased from day 9 pi (Fig. 5E).

## DISCUSSION

Consistent with a previous study, the main symptom of intranasal SARS-CoV-2 infection in Syrian hamsters was weight loss and recovery without obvious sequelae (8–10, 14). Our data demonstrated that weight change can be separated into two stages, namely, the acute onset of rapid weight loss and the recovery course of gradual weight recovery. At the acute onset, we found that the gRNA and sgRNA of the virus were present in the lungs of Syrian hamsters, and the copy number of sgRNA was lower than that of gRNA (approximately 5% of these genes). Previous studies found that, although sgRNA test is negative, the gRNA test is still positive during the recovery period or after an effective treatment (15, 16). Therefore, it is likely that the detection of sgRNA shows the effect of vaccines, monoclonal antibodies, or other interventions on SARS-CoV-2 replication in hamster models.

In this study, we selected male hamsters of 8 to 10 weeks of age that were in the stage equivalent to human puberty (17). At this stage, a human year is equivalent to 3.65 days in a hamster. Therefore, we continued to observe the pathological changes for 20 days after infection in hamsters, equivalent to the long-term changes of 5 years after infection in humans. Virus infection dose affects the lung inflammation severity and weight loss in a hamster model (8, 9, 11, 14). As in previous studies, 10^5^ PFU SARS-CoV-2-infected hamsters were used to study their pathological changes and the effects on blood chemistry. We observed that the histopathology of the infected hamsters showed severe lung lesions and inflammation at day 5 pi However, Masson’s staining revealed that the state of collagen fiber deposition in the lesion area lasted until day 20. These observations indicate that SARS-CoV-2 can cause severe lung damage in the early stages of infection and continue to evolve into pulmonary fibrosis during the recovery period. Previous studies have pointed out that dyspnea and cough caused by fibrotic lung injury are the most common daily conditions for survivors of COVID-19 (5, 18). Therefore, further investigation is required to determine whether hamsters can be used as a research model for long-term COVID syndrome.

Many biochemical parameters are altered in COVID-19 patients, which correlates with disease severity (19–22). The biochemical parameters with increased levels in these severe COVID-19 patients include alanine transaminase (ALT), aspartate transaminase (AST), blood urea nitrogen (BUN), bilirubin, urea, creatine, amylase, lipase, procalcitonin, C-reactive protein (CRP), D-dimer, and lactate dehydrogenase (LDH). The parameters with decreased levels were total protein and albumin. When disease recovered, creatine, glucose, ALT, AST, potassium, and LDH levels remained above reference range in more than 10% of patients (23). In addition to our study, other literature has also analyzed biochemical markers from SARS-CoV-2-infected hamsters (14, 24). Small increases in the measurements of amylase, lipase, and ALT were found in hamsters similar to those observed in COVID-19 patients. However, the trends of other biochemical parameters, such as AST, BUN, bilirubin, urea, creatine, and LDH, were different between humans and hamsters. The different findings between humans and hamsters may reflect that SARS-CoV-2 virus tropism depends on the susceptibility and permissiveness in the host. In our study, we designed a case-control experiment in a hamster model and continuously monitored serum biochemical markers. The dynamic trends of biochemical parameters in blood after SARS-CoV-2 infection are clearly visible, such as of those of lipid metabolism. Taken together, both human and hamster blood analysis data suggest some dysregulation in extrapulmonary organs and may indicate acute sequelae of SARS-CoV-2.

SARS-CoV-2 has been confirmed in animals and humans and can cause dysfunction of various organs, including the lungs (11, 13, 19). Abnormal liver function was a significant observation in patients with COVID-19 (20, 21). A previous study on infected hamsters found that the AST/ALT ratio increased at acute onset, accompanied by structural abnormalities and large vacuoles in the liver (14). However, we found that the AST of the infection group increased slightly during the long-term monitoring, compared with that of the control group, while other hepatitis-related indicators did not change significantly. ALB levels also decreased significantly in patients with severe COVID-19 (22). In our study, ALB levels decreased significantly during the recovery process of infected hamsters, similar to a previous study showing hypoalbuminemia in Nipah virus-infected hamsters (23). We believe that the low serum protein levels of hamsters after viral infection may be due to malnutrition caused by anorexia or critical illness, rather than liver failure. Based on our findings and previous studies, we believe that the effect of SARS-CoV-2 on the liver is worthy of further research to verify the hamster model.

Low serum UA levels are common in patients with COVID-19 and are related to disease severity (24, 25). Surprisingly, we found that the UA level of infected hamsters was significantly higher than that of the control group. Previous studies have found that mice or children infected with respiratory syncytial virus (RSV) have significantly increased UA levels in bronchiole lavage fluid (26). Increased UA production has a significant impact on RSV immunopathology associated with the cytokines interleukin-1β (IL-1β) and IL-33 in the lungs. Moreover, serum creatine (CREA) levels significantly reduced during the recovery period. Low CREA levels may indicate muscle wasting, such as decreased muscle mass in the elderly (27), suggesting an underlying mechanism to help hamsters recover quickly after infection and cause high UA and low CREA levels.

Several studies have reported important changes in the blood lipid profile of patients with COVID-19, including decreased cholesterol and high-density lipoprotein (HDL) levels and increased triglycerides (28–30). Our results showed that triglycerides in the infected group increased significantly at day 3 pi and then recovered at day 9 pi Total cholesterol (T-CHOL) and HDL levels decreased at day 5 pi and recovered at day 9 pi The changes in blood lipids of Syrian hamsters at the initial stage of infection were consistent with the clinical COVID-19 patient survey (28–30). However, we found that LDL levels increased during the recovery course. These results suggest that lipid metabolism may play an important role in the survival and recovery after infection and require further investigation.

To our knowledge, this is the first report on the use of serially collected blood samples from a SARS-CoV-2-infected hamster model to analyze the dynamic changes in the blood biochemistry during infection. Our data showed that blood lipid, uric acid, creatine, total protein, and albumin levels were related to disease progression. In addition, we confirmed that SARS-CoV-2 infection has long-term effects on hamsters. Therefore, this model may be utilized to explore the causes and effects of long-COVID syndrome.

## MATERIALS AND METHODS

### Virus and cell culture.

All studies involving live SARS-CoV-2 were performed in Taiwan Centers for Disease Control Prevention-approved biosafety level 3 facility and animal biosafety level 3 laboratory at the Institute of Preventive Medicine (IPM). SARS-CoV-2 (human/TWN/CGMH-CGU-04/2020; GenBank number MT370517) was isolated from a confirmed COVID-19 patient originally from Wuhan (China) on March 2020 in Taiwan (31). SARS-CoV-2 was inoculated into Vero E6 cells and stored at −80°C in single-use aliquots. Viral titers were determined using a plaque assay, as described previously (10).

### Hamster experiments.

The protocol was approved by the Institutional Animal Care and Use Committee of IPM. Male Syrian hamsters (*Mesocricetus auratus*) aged 8 to 10 weeks old on study initiation were purchased from the National Laboratory Animal Center (Taipei, Taiwan). All hamsters were randomly distributed into two groups: mock (*n* = 11) and SARS-CoV-2 infected (*n* = 11). Hamsters were anesthetized via deep intramuscular injection with Zoletil 50 and xylazine (Anesedan1, Brazil) at doses of 200 mg/kg and 10 mg/kg, respectively. Micro-transponder (Lifechip, Destron Fearing, Saint Paul, USA) was subcutaneously implanted into all hamsters 2 days prior to infection for identification. Animals were mock infected with 100 µL phosphate-buffered saline (PBS) or infected with 10^5^ PFU SARS-CoV-2 in 100 µL through intranasal instillation. On days 5 and 20 postinfection (pi), three randomly assigned hamsters from each group were euthanized via exsanguination. Half of the lung was used to determine viral load (virus titration) and genome amount (real-time quantitative PCR; RT-qPCR). The other half of the lung was fixed in 4% formalin for histopathological examination. During the 20-day experiment, body weight, clinical signs, and survival were measured daily. Serial blood samples from the cranial vena cava were collected 2 days prior to infection and on days 3, 4, 10, 15, and 20 pi for biochemical analysis. Blood (≤6 mL) was collected at each time point.

### Virus titration.

For the assessment of virus titers from lung tissue, tissue homogenates were serially diluted in Dulbecco’s modified Eagle medium (DMEM), and plaque assay was performed. The diluent was seeded on Vero E6 cells and incubated with DMEM containing 0.3% agarose (Invitrogen, USA) at 37°C, 5% CO_2_. The cytopathic effect was observed daily until the appearance of single plaques. The plaque number per milliliter (PFU/mL) was counted after fixation of the cells with 4% formaldehyde and staining with 0.2% crystal violet to clearly visualize single plaques.

### RNA extraction and RT-qPCR.

Total RNA was extracted from infected cells using TRIzol reagent (Thermo Fisher Scientific, USA). The cDNA was synthesized using the SuperScript IV first-strand synthesis system (Thermo Fisher Scientific). The transcription levels of the SARS-CoV-2 nucleocapsid (NP), subgenomic envelope gene, envelope (E) gene, ORF1ab region, and hamster glyceraldehyde-3-phosphate dehydrogenase gene (*GAPDH*) were measured using RT-qPCR in the LightCycler 480 II instrument (Roche). All primers used are listed in Table 1. For quantification of gene expression, the genes were cloned into a pGEM-T easy vector (Promega). The plasmid was then used as a template for PCR amplification using the T7 or SP6 primers. The PCR products were quantified using NanoDrop (Thermo Fisher Scientific) and used as templates for the standard curve generated from serial dilutions of PCR products (1 to 10^6^ copies/reaction [Rn]). The SARS-CoV-2 and *GAPDH* copy numbers in the sample were assessed by comparing sample threshold cycle (*C_T_*) values to a standard curve and expressed in log_10_ copies of SARS-CoV-2 virus per 10^5^ copies of *GAPDH*.

**TABLE 1:** Primers used in this study

Primer name	Sequence (5′ to 3′)	Feature/purpose
SARSCoV2_NP_F	GCCTCTTCTCGTTCCTCATCAC	RT-PCR of SARS-CoV-2 nucleocapsid (NP) gene
SARSCoV2_NP_R	AGCAGCATCACCGCCATTG	
SARSCoV2_SubE_F	CGATCTCTTGTAGATCTGTTCTC	RT-PCR of SARS-CoV-2 subgenomic envelope (E) gene
SARSCoV2_SubE_R	ATATTGCAGCAGTACGCACACA	
SARSCoV2_E_F	ACAGGTACGTTAATAGTTAATAGCGT	RT-PCR of SARS-CoV-2 envelope (E) gene
SARSCoV2_E_R	ATATTGCAGCAGTACGCACACA	
SARSCoV2_ORF1ab_F	CCCTGTGGGTTTTACACTTAA	RT-PCR of ORF1ab region
SARSCoV2_ORF1ab_R	ACGATTGTGCATCAGCTGA	
Hamster_G3pdh_F	GACATCAAGAAGGTGGTGAAGC	RT-PCR of hamster GAPDH gene
Hamster_G3pdh_R	CATCAAAGGTGGAAGAGTGGGA	

### Histopathological examinations.

Lung tissues were fixed in 4% paraformaldehyde for 48 h, and paraffin sections (4 μm in thickness) were applied following the routine operating procedure. Hematoxylin and eosin (H&E) and Masson’s trichrome stains (Masson) were used to identify histopathological changes in hamster lungs. Immunohistochemical staining was performed to detect the NP using a rabbit polyclonal SARS-CoV-2 (GeneTex, GTX135357) at a dilution of 1:1,000. To observe the overview of whole lung lobe sections, all slides were scanned using a slide scanner.

### Blood biochemical analysis.

To evaluate liver function, kidney function, lipoprotein profiles, metabolism panels, and mineral elements during the course of infection, hamster serum was analyzed using an automated chemical analyzer (Hitachi 7080, Hitachi High-Technologies Corporation) for 20 serum biochemical parameters. Samples with visible hemolysis, lipemia, or icteric discoloration were excluded from biochemical analysis. Parameters related to liver function were aspartate transaminase (AST), alanine transaminase (ALT), total bilirubin (T-BIL), gamma-glutamyl transpeptidase (GGT), alkaline phosphatase (ALP), and lactate dehydrogenase (LDH). Blood urea nitrogen (BUN), creatinine (CREA), and uric acid (UA) were used to assess kidney function. The lipoprotein profiles included triglyceride (TG), total cholesterol (T-CHOL), high-density lipoprotein (HDL), and low-density lipoprotein (LDL). Other metabolic panels included total protein (TP), albumin (ALB), glucose (GLU), and creatine phosphokinase (CPK). The mineral elements included magnesium (Mg), calcium (Ca), and potassium.

### Data analysis.

Blood biochemical data were expressed as the mean ± standard deviation. Data analysis was performed with GraphPad Prism software using the Mann–Whitney test. (GraphPad software, La Jolla, CA, USA). Differences in the biochemical markers between uninfected and infected animals were considered statistically significant at *P* < 0.05.
